# Development of a risk assessment model for the recurrence of high-grade squamous intraepithelial lesions and differentiated vulvar intraepithelial neoplasia in Chinese cohort

**DOI:** 10.1007/s12672-026-04714-w

**Published:** 2026-02-19

**Authors:** Xi Ye, Xiangfeng Zhang, Le Yu, Chenmin Zheng, Xuanxuan Hong, Fei Wang, Liehong Wang

**Affiliations:** 1 Qinghai University Affiliated Clinical Medical College, Xining, Qinghai China; 2 Obstetrics and Gynecology Department, Anhui Province Maternal and Child Health Hospital, Hefei, Anhui China; 3 Qinghai Red Cross Hospital, 55 South Street, Xining, Qinghai China

**Keywords:** High-grade squamous intraepithelial lesions of the vulva, Differentiated vulvar disease, Recurrence, Regression analysis, Prediction model

## Abstract

**Objective:**

To construct a sophisticated risk prediction model for the recurrence of high-grade squamous intraepithelial lesions (HSIL) and differentiated vulvar intraepithelial neoplasia (dVIN) after treatment. This model will help the early detection and focused screening of individuals at increased risk of dVIN.

**Methods:**

The clinical data from 257 patients diagnosed with dVIN or HSIL were retrospectively reviewed. Patients were divided into two distinct cohorts: relapse (*n* = 60) and non-recurrence (*n* = 197). For robust model development, the dataset was methodically divided into two subsets: training (70% of cases) and validation (30% of cases). Logistic regression analysis was applied to identify key predictors. Subsequently, they were combined to construct a risk prediction model for post-treatment recurrence of HSIL and dVIN.

**Results:**

Univariate logistic regression analysis revealed that age, menopause, immunosuppression, HPV16 infection, histopathological characteristics, and positive surgical margins were positively correlated with recurrence risk. In contrast, low-risk HPV types exhibited a negative correlation with recurrence risk (all *P* < 0.05). Multivariable logistic regression analysis revealed that age, smoking history, immunosuppression, HPV16 infection, and histopathology were robustly associated with an increased recurrence risk (all *P* < 0.05). In the training dataset, the area under the receiver operating characteristic curve (AUC) was 0.793 (95% CI 0.77–0.880), accompanied by a median prediction success probability of 0.810. In the internal validation dataset, the AUC improved to 0.831 (95% CI 0.726–0.937). The Hosmer–Lemeshow goodness-of-fit test revealed acceptable model calibration for the training (*P* = 0.069) and internal validation (*P* = 0.086) sets. Calibration curves revealed trends remarkably consistent with the ideal curve, indicating commendable calibration. Furthermore, clinical decision curve analysis substantiated the net benefit of the model.

**Conclusion:**

A prediction model including age, smoking history, immunosuppression, HPV16 infection, and histopathology exhibits good predictive performance for post-treatment recurrence of HSIL and dVIN. Our model can help clinicians assess recurrence risk, providing guidance for clinical consultations and enabling targeted follow-up and treatment plans for individuals at high risk.

## Introduction

Vulvar cancer is among the most common malignancies of the lower reproductive tract in postmenopausal women, accounting for approximately 4% of female genital tract malignancies. In the United States, 6330 new cases are diagnosed annually, resulting in 1560 deaths [1]. While the exact cause of vulvar carcinoma remains unclear, vulvar squamous cell carcinoma (VSCC) often develops from vulvar intraepithelial neoplasia (VIN). Current evidence suggests VSCC arises via two distinct pathways: one linked to human papillomavirus (HPV), involving high-grade squamous intraepithelial lesions (HSIL), and another HPV-independent pathway, associated with differentiated VIN (dVIN), which typically occurs alongside vulvar lichen sclerosus (VLS), a non-neoplastic vulvar disorder [2, 3]. The World Health Organization’s 2020 classification divides vulvar lesions into HPV-related (including LSIL and HSIL) and non-HPV-related (encompassing dVIN, differentiated exophytic lesions, and vulvar acanthosis with altered differentiation [VAAD]) [4]. Although vulvar precancerous lesions are relatively rare, their incidence has been rising [5–7]. Early-stage disease often lacks specific symptoms, occasionally presenting with mild vulvar pruritus, while some patients remain asymptomatic, delaying diagnosis and potentially leading to concurrent early invasive squamous cell carcinoma [8]. No effective screening methods exist for vulvar precancerous lesions; diagnosis relies on integrating medical history and clinical findings, with pathological examination as the gold standard. However, diagnostic consistency for HSIL and dVIN is low, often requiring immunohistochemical analysis for accurate classification [9].

For vulvar LSIL, observation is recommended due to its high natural resolution rate. In contrast, vulvar HSIL and dVIN require intervention and standardized management due to their high progression risk [10]. Treatment options include surgery (preferring conservative or minimally invasive approaches to preserve vulvar function [11]), pharmacotherapy (e.g., imiquimod used for HPV-related HSIL and topical cidofovir [12, 13]. Physical therapy primarily comprises CO_2_ laser vaporization and localized photodynamic therapy [14, 15]. However, regardless of whether surgical, pharmacological, or physical therapies are employed, all treatment modalities exhibit a relatively high recurrence rate [16], with a lifelong risk of recurrence or malignant transformation, particularly in patients with high-risk factors such as multifocal lesions, lesion size of > 2 cm, positive surgical margins, persistent high-risk HPV infection, VLS, advanced age, and immunosuppression [17]. Subsequently, in the present study, we identified the risk factors underlying dVIN and vulvar HSIL, using clinical data to develop a sophisticated prediction model for the post-treatment recurrence of these conditions. We aimed to promptly identify high-risk groups during follow-up and implement focused monitoring and rigorous follow-up protocols, thereby minimizing the probability of disease recurrence or progression.

## Data and methods

### Clinical information

The clinical data of 257 patients with dVIN and vulvar HSIL who received treatment and participated in regular follow-ups at Anhui Provincial Maternal and Child Health Hospital between January 2010 and January 2025 were retrospectively reviewed. Based on whether the patients experienced recurrence after treatment, the patients were divided into two groups: relapse (comprising 60 individuals) and non-recurrence (comprising 197 individuals). The diagnostic criterion was the pathological diagnosis of dVIN and vulvar HSIL. The pathological diagnosis was confirmed via consensus from at least two experienced pathologists.

#### Inclusion criteria

(1) Confirmed pathological diagnosis of dVIN or vulvar HSIL; (2) HPV detection via exfoliated cytological examination at the sampling site; (3) patients who received treatment and were followed up for at least 2 years after diagnosis; (4) those with complete, accurate, and reliable clinical data records, including related symptoms, physical examination findings, and auxiliary examination results; and (5) those who voluntarily signed the informed consent form for clinical data investigation and follow-up.

#### Exclusion criteria

(1) Pathological diagnosis revealed other VIN types; (2) pathological diagnosis confirmed invasion of other malignant tumors into the vulva; (3) pathological diagnosis confirmed other vulvar lesions; (4) exfoliated cytological HPV detection was not performed at the sampling site; (5) combined pregnancy; (6) incomplete clinical data or recording errors; and (7) irregular follow-up or loss to follow-up.

The study was conducted according to the ethical guidelines of the 2013 version of the Declaration of Helsinki. It was approved by the hospital’s Ethics Committee. Informed consent was voluntarily obtained from both the patients and their families, who fully understood the details of the study.

### Research method

#### Candidate predictor

According to the literature, the candidate variables were easily obtainable clinical, pathological, and laboratory indicators. These factors were age, body mass index (BMI), menopausal status, family history of cancer, history of smoking, history of alcohol consumption, history of immunosuppressant use, comorbidities (e.g., benign hypertension, type 2 diabetes, autoimmune diseases), HPV test results (e.g., type 16, high-risk types other than type 16, low-risk types), presence of cervical lesions, presence of vulvar pruritus, presence of visible vulvar lesions (e.g., ulcers, flat warts, degeneration), whether a total hysterectomy was performed, number of lesions (single or multiple), lesion size (mean diameter), histopathological findings, immunohistochemical markers (e.g., p53, Ki67), and treatment modality (e.g., surgical, pharmacological, physical). For surgical treatment, the variable was the presence of a positive surgical margin. For the pathological diagnosis of HSIL, the variable was whether interferon was chosen as adjuvant therapy.

#### Observation index

Follow-up information for the patients was obtained through telephonic consultations, regular outpatient visits, and other methods. Considering that the disease requires lifelong follow-up owing to its potential for recurrence or progression, follow-ups were conducted every 6–12 months. The study included patients with at least 2 years of follow-up data; these patients were categorized into either the relapse or non-recurrence group, depending on whether their condition recurred or worsened(It is accurately defined as new preinvasive VIN/HSIL/dVIN lesion or progression to invasive VSCC).

#### Statistical analysis

SPSS 25.0 and R 4.4.2 software were used to conduct statistical analyses. If continuous variables adhered to a normal distribution, the mean ± standard deviation was utilized for statistical description. The independent sample t-test was applied for comparisons between groups. In contrast, for data that did not conform to a normal distribution, the median [P25, P75] was reported. The rank sum test was conducted for group comparisons. The chi-squared test was used for comparing categorical variables across groups. In cases in which the assumptions of the chi-squared test were not satisfied, Fisher’s exact probability test was applied to identify risk factors. Subsequently, R 4.4.2 software was used to develop a prediction model and generate nomogram charts. Receiver operating characteristic (ROC) curves were used to predict the discriminatory power of the prediction model. Graphical calibration methods and the Hosmer–Lemeshow goodness-of-fit test were used to assess the calibration performance. Finally, decision curve analysis (DCA) was performed to evaluate the clinical applicability of the prediction model. The statistical significance level was set at *p* < 0.05.

#### Prediction model construction

Univariate logistic regression analysis was used to identify potential predictors of outcome events (*P* < 0.1). Thereafter, the least absolute shrinkage and selection operator (LASSO) logistic regression method was applied to define the number of selected variables, identifying the key features that exhibit non-zero coefficients. The best parameter settings were identified using 10-fold cross-validation, and the coefficients were computed based on the lambda value (min) with the least deviation. Variables with non-zero coefficients were used for additional analysis. Subsequently, the selected variables were subjected to a stepwise, bidirectional multivariate logistic regression. Then, a detailed nomogram was developed for the prediction model, utilizing the variables identified via the stepwise method with a significance level of *P* < 0.05. For internal validation, bootstrapping with 1000 resamples was conducted. Calibration curves were constructed to thoroughly assess the model’s calibration performance, whereas the Hosmer–Lemeshow test was employed to evaluate its goodness of fit. ROC curves were analyzed to evaluate key metrics, including the area under the curve (AUC), sensitivity, and specificity. In addition, clinical DCA was applied to thoroughly assess the model’s applicability in clinical settings and to measure the net benefit across various threshold probabilities. Finally, the developed model underwent external validation using the validation set.

## Results

### Clinical characteristics of the sample cohort

In total, 257 patients were enrolled, with 60 patients in the relapse group; this corresponded to an incidence rate of 23.35% (60/257). Among the cohort, 58 cases were diagnosed with newly developed preinvasive VIN/HSIL/dVIN lesions. The mean time to first recurrence following surgical intervention was 2.88 ± 1.33 years, with an average recurrence count of 2.00 ± 0.82 episodes per patient. Upon administration of the aforementioned therapeutic modalities—surgical excision, pharmacological treatment, and physical therapy—and continuous follow-up to the predetermined cutoff date of January 2025, no instances of disease progression to invasive vulvar squamous cell carcinoma (VSCC) were observed in the majority of cases. Notably, however, two isolated cases did exhibit malignant transformation into invasive VSCC, occurring specifically during the second and fourth recurrences, respectively. Importantly, postoperative pathological assessments revealed no evidence of invasive carcinoma at the time of surgery, and throughout the entire follow-up period up to January 2025, no further development of invasive cancer was detected. The dataset was methodically divided into two subsets: training, accounting for 70% of the cases, and validation, accounting for the remaining 30%, in a 7:3 ratio. The training set served as the foundation for developing the model, whereas the validation set was used for verifying the model. The results are summarized in Table 1 (Baseline table description of the training and validation sets).

**Table 1:** Baseline Table: Description of the Training Set and Validation Set

Variables	Total (*n* = 257)	Training (*n* = 179)	Validation (*n* = 78)	*p*
Age, Median (Q1,Q3)	54.00 (47.00, 63.00)	53.00 (47.00, 63.00)	55.50 (48.00, 63.00)	0.479
BMI, Median (Q1,Q3)	24.51 (21.56, 26.83)	24.57 (21.87, 27.14)	23.63 (21.28, 26.57)	0.264
Menopause, n (%)	145 (56.4)	99 (55.3)	46 (59.0)	0.586
Family history, n (%)	30 (11.7)	26 (14.5)	4 (5.1)	0.031
Smoking history, n (%)	67 (26.1)	49 (27.4)	18 (23.1)	0.471
Drinking history, n (%)	48 (18.7)	35 (19.6)	13 (16.7)	0.585
immunosuppressant, n (%)	50 (19.5)	41 (22.9)	9 (11.5)	0.034
Hypertension, n (%)	67 (26.1)	42 (23.5)	25 (32.1)	0.149
Type 2 diabetes, n (%)	22 (8.6)	14 (7.8)	8 (10.3)	0.521
Autoimmunedisease, n (%)	20 (7.8)	14 (7.8)	6 (7.7)	0.972
HPV16, n (%)				0.106
Negative	205 (79.8)	138 (77.1)	67 (85.9)	
Positive	52 (20.2)	41 (22.9)	11 (14.1)	
Other high-risk HPV types, n (%)				0.364
Negative	194 (75.5)	138 (77.1)	56 (71.8)	
Positive	63 (24.5)	41 (22.9)	22 (28.2)	
Low-risk HPV, n (%)				0.028
Negative	206 (80.2)	137 (76.5)	69 (88.5)	
Positive	51 (19.8)	42 (23.5)	9 (11.5)	
Cervical lesions, n (%)	70 (27.2)	54 (30.2)	16 (20.5)	0.110
Pruritus vulvae, n (%)	54 (21.0)	40 (22.3)	14 (17.9)	0.426
Lesions of the vulva, n (%)	34 (13.2)	23 (12.8)	11 (14.1)	0.785
Total hysterectomy, n (%)				0.931
Have	17 (6.6)	12 (6.7)	5 (6.4)	
None	240 (93.4)	167 (93.3)	73 (93.6)	
Number of lesions, n (%)				0.946
Multiple	197 (76.7)	137 (76.5)	60 (76.9)	
Single	60 (23.3)	42 (23.5)	18 (23.1)	
Focal size, Median (Q1,Q3)	1.70 (1.40, 2.20)	1.70 (1.35, 2.20)	1.80 (1.50, 2.00)	0.689
Histopathology, n (%)				0.033
dVIN	35 (13.6)	19 (10.6)	16 (20.5)	
HSIL	222 (86.4)	160 (89.4)	62 (79.5)	
p53, n (%)				0.424
Negative	233 (90.7)	164 (91.6)	69 (88.5)	
Positive	24 (9.3)	15 (8.4)	9 (11.5)	
Ki67, Median (Q1,Q3)	40.00 (20.00, 60.00)	45.00 (20.00, 65.00)	40.00 (20.00, 60.00)	0.404
Operation, n (%)	73 (28.4)	55 (30.7)	18 (23.1)	0.211
Drug therapy, n (%)	70 (27.2)	52 (29.1)	18 (23.1)	0.323
Physical therapy, n (%)	44 (17.1)	25 (14.0)	19 (24.4)	0.042
Incisal positive, n (%)	18 (7.0)	15 (8.4)	3 (3.8)	0.190
Interferon, n (%)	86 (33.5)	67 (37.4)	19 (24.4)	0.041

dVIN: differentiated vulvar intraepithelial neoplasia

HSIL: vulvar high-grade squamous intraepithelial lesions

Chi-square tests were used for categorical variable comparisons across groups

Hypertension and Type II diabetes are confirmed with lab tests according to clinical criteria

### Post-treatment recurrence analysis of HSIL and differentiated vulvar lesions

**Fig. 1:**
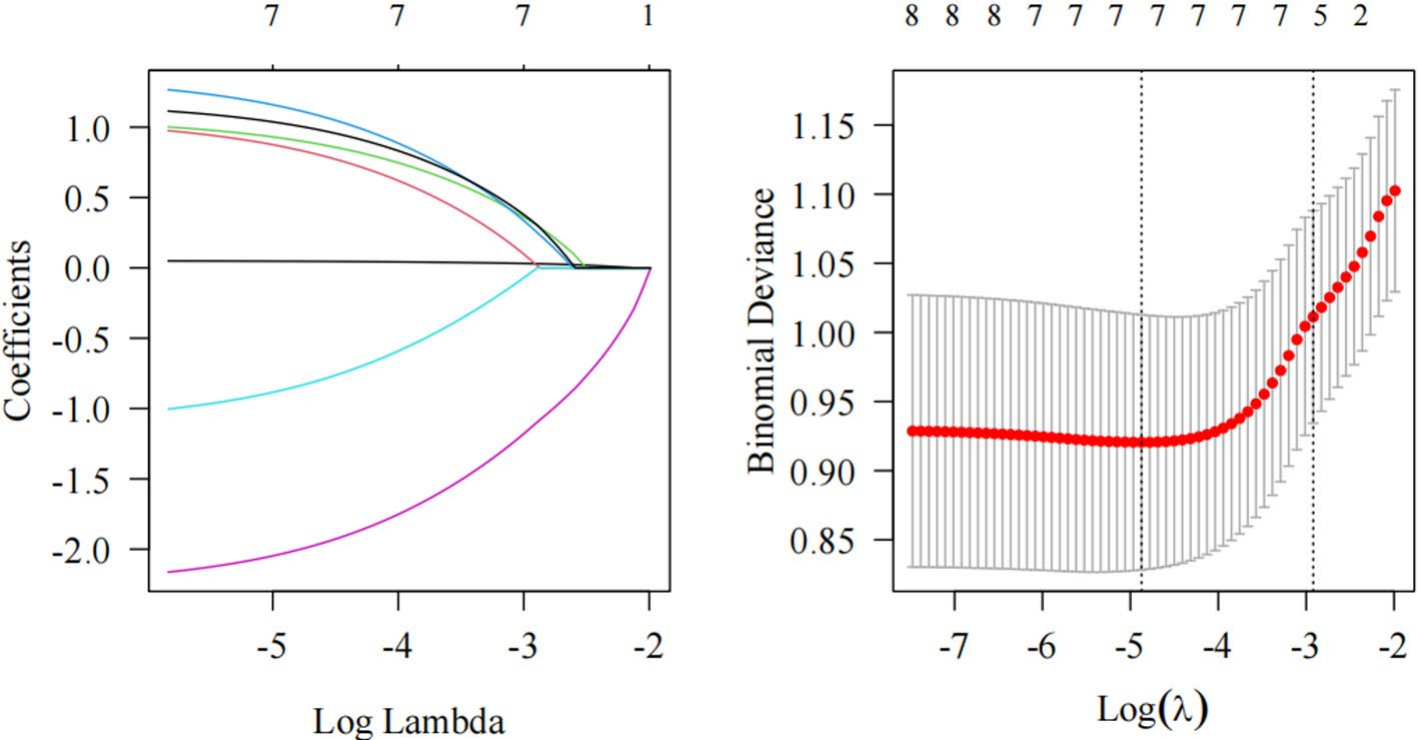
Lasso regression and error bar plot

Univariate logistic analysis identified the risk factors for recurrence. Age, menopause, immunosuppression, HPV16 infection, histopathological findings, and positive surgical margins were positively correlated with the risk of recurrence. However, low-risk HPV infection was negatively correlated with the risk of recurrence (all *P* < 0.05). The regression coefficient (β), standard error (SE), Z-value, P-value, odds ratio (OR), and corresponding 95% confidence interval (CI) for the variables associated with the post-treatment recurrence of HSIL and differentiated vulvar lesions are systematically outlined. Specific results are presented in Table 2 (single-factor logistic regression results of the training set). To account for multicollinearity among variables, variables exhibiting *P* < 0.1 in the univariate logistic regression analysis were incorporated into the LASSO regression model. Variables with non-zero coefficients were identified based on their lambda value (0.008) corresponding to the minimum deviation plus one SE. Table 3 (LASSO screening coefficient variables) and Fig. 1 (LASSO regression and error bar plot) summarize the comprehensive findings. In the training dataset, the significant variables determined via univariate analysis were subjected to multivariate logistic regression analysis. Analysis revealed that age, a history of smoking, immunosuppression, HPV16 infection, and histopathological characteristics(HSIL) were positively correlated with the risk of recurrence (all *P* < 0.05). Table 4 (Multivariate logistic regression results of the training set) presents the precise outcomes.

**Table 2:** Single factor logistic regression results of training set

Variable	β	SE	OR(95%CI)	*p*
Age	0.073	0.020	1.075 (1.036, 1.119)	< 0.001
BMI	0.027	0.056	1.027 (0.920, 1.148)	0.635
Menopause	1.051	0.390	2.860 (1.364, 6.377)	0.007
Family history	−0.292	0.532	0.746 (0.236, 1.983)	0.583
Smoking history	0.662	0.375	1.939 (0.919, 4.032)	0.078
Drinking history	−0.250	0.465	0.779 (0.292, 1.855)	0.590
immunosuppressant	1.014	0.387	2.757 (1.280, 5.887)	0.009
Hypertension	−0.151	0.426	0.860 (0.356, 1.922)	0.722
Type 2 diabetes	−0.127	0.677	0.881 (0.192, 2.992)	0.852
Autoimmunedisease	0.290	0.620	1.337 (0.351, 4.250)	0.640
HPV16				
Negative	0.000		reference	
Positive	0.864	0.389	2.372 (1.094, 5.066)	0.026
Other high-risk HPV types				
Negative	0.000		reference	
Positive	0.399	0.401	1.490 (0.663, 3.221)	0.320
Low-risk HPV				
Negative	0.000		reference	
Positive	−1.007	0.514	0.365 (0.119, 0.926)	0.050
Cervical lesions	−0.254	0.396	0.776 (0.345, 1.650)	0.522
Pruritus vulvae	0.443	0.402	1.557 (0.691, 3.378)	0.271
Lesions of the vulva	−0.113	0.539	0.893 (0.280, 2.418)	0.834
Total hysterectomy				
Have	0.000		reference	
None	0.454	0.796	1.575 (0.394, 10.522)	0.568
Number of lesions				
Multiple	0.000		reference	
Single	−0.339	0.440	0.713 (0.284, 1.626)	0.442
Focal size	0.444	0.286	1.559 (0.885, 2.738)	0.121
Histopathology				
dVIN	0.000		reference	
HSIL	−2.005	0.517	0.135 (0.047, 0.363)	< 0.001
p53				
Negative	0.000		reference	
Positive	0.187	0.613	1.206 (0.320, 3.756)	0.760
Ki67	0.008	0.007	1.008 (0.995, 1.022)	0.224
Operation	−0.134	0.388	0.874 (0.397, 1.837)	0.729
Medicine	0.546	0.372	1.726 (0.822, 3.566)	0.142
Physical therapy	−0.542	0.577	0.581 (0.162, 1.645)	0.347
Incisal positive	1.475	0.552	4.370 (1.471, 13.294)	0.008
Interferon	−0.233	0.371	0.792 (0.375, 1.620)	0.531

dVIN: differentiated vulvar intraepithelial neoplasia

HSIL: vulvar high-grade squamous intraepithelial lesions

Using univariate logistic regression analysis to evaluate potential predictors for outcome events were carefully evaluated

Univariate logistic analysis results indicated that age, menopause, immunosuppression, HPV16 infection, histopathological findings, and positive surgical margins were positively correlated with the risk of recurrence, whereas low-risk HPV infection was negatively correlated with recurrence risk

**Table 3:** Lasso screening coefficient variable

Trait	coefficient	lambda.min
(Intercept)	−3.912	0.008
Age	0.0471	
Smoking history	0.853	
Immunosuppressant	0.915	
HPV16	1.136	
Low-risk HPV	−0.858	
Histopathology	−2.022	
Incisal positive	1.020	

The least absolute shrinkage and selection operator (LASSO) logistic regression method was applied to narrow down the number of selected variables

**Table 4:** Multivariate logistic regression results of training set

Variable	β	SE	OR(95%CI)	*p*
Age	0.051	0.022	1.052 (1.008,1.100)	0.022
Smoking history	1.059	0.466	2.884 (1.167,7.364)	0.023
Immunosuppressant	1.060	0.453	2.886 (1.185,7.090)	0.019
HPV16				
Negative	0.000		reference	
Positive	1.356	0.485	3.882 (1.516,10.319)	0.005
Low-risk HPV				
Negative	0.000		reference	
Positive	−1.106	0.627	0.331 (0.085,1.035)	0.078
Histopathology				
dVIN	0.000		reference	
HSIL	−2.260	0.606	0.104 (0.030,0.330)	< 0.001
Incisal positive	1.177	0.663	3.245 (0.877,12.189)	0.076

dVIN: differentiated vulvar intraepithelial neoplasia

HSIL: vulvar high-grade squamous intraepithelial lesions

A stepwise, bidirectional multivariate logistic regression was used to identify factor for prediction model

### Nomogram for predicting the post-treatment recurrence of HSIL and differentiated vulvar lesions

**Fig. 2:**
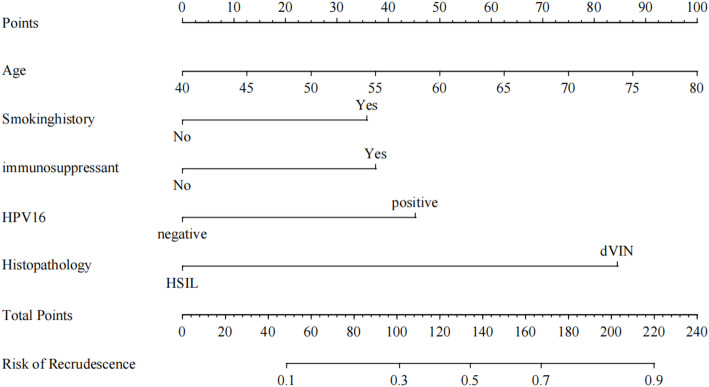
Nomograph

RGui software was used to include the five predictors identified via multivariate regression analysis for nomogram construction, as illustrated in Fig. 2 (Nomogram). In the figure, the first row represents the score corresponding to each variable. The score value for each variable was obtained by drawing a vertical line upward. The overall score was calculated by aggregating the scores of all variables. Positioned in the penultimate row, this total score served as a reference point from which a vertical line was drawn down to the last row, thereby indicating the associated predicted risk probability for the outcome.

### ROC curve analysis for predicting the post-treatment recurrence of HSIL and differentiated vulvar lesions

**Fig. 3:**
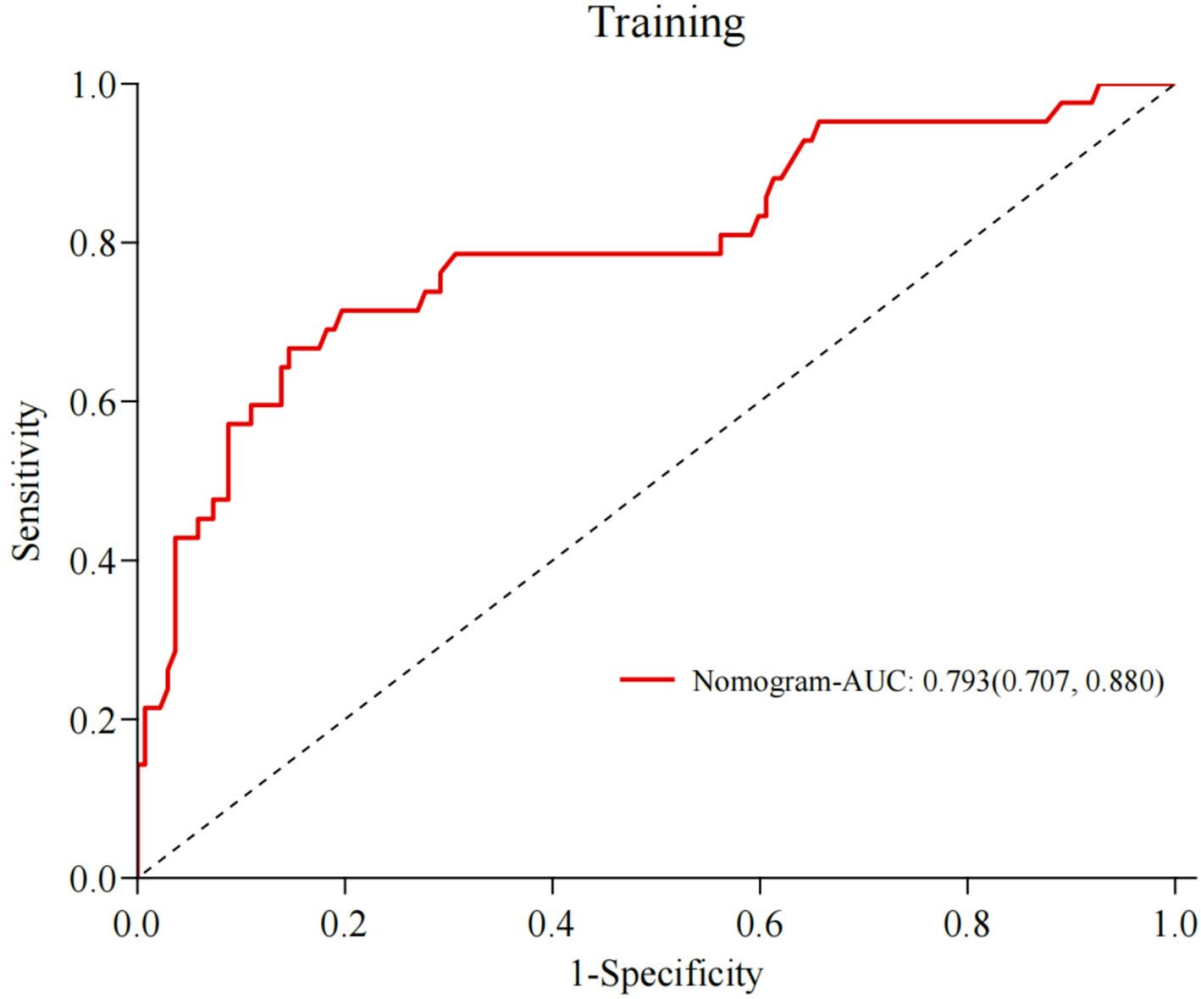
ROC curves for the training set

**Fig. 4:**
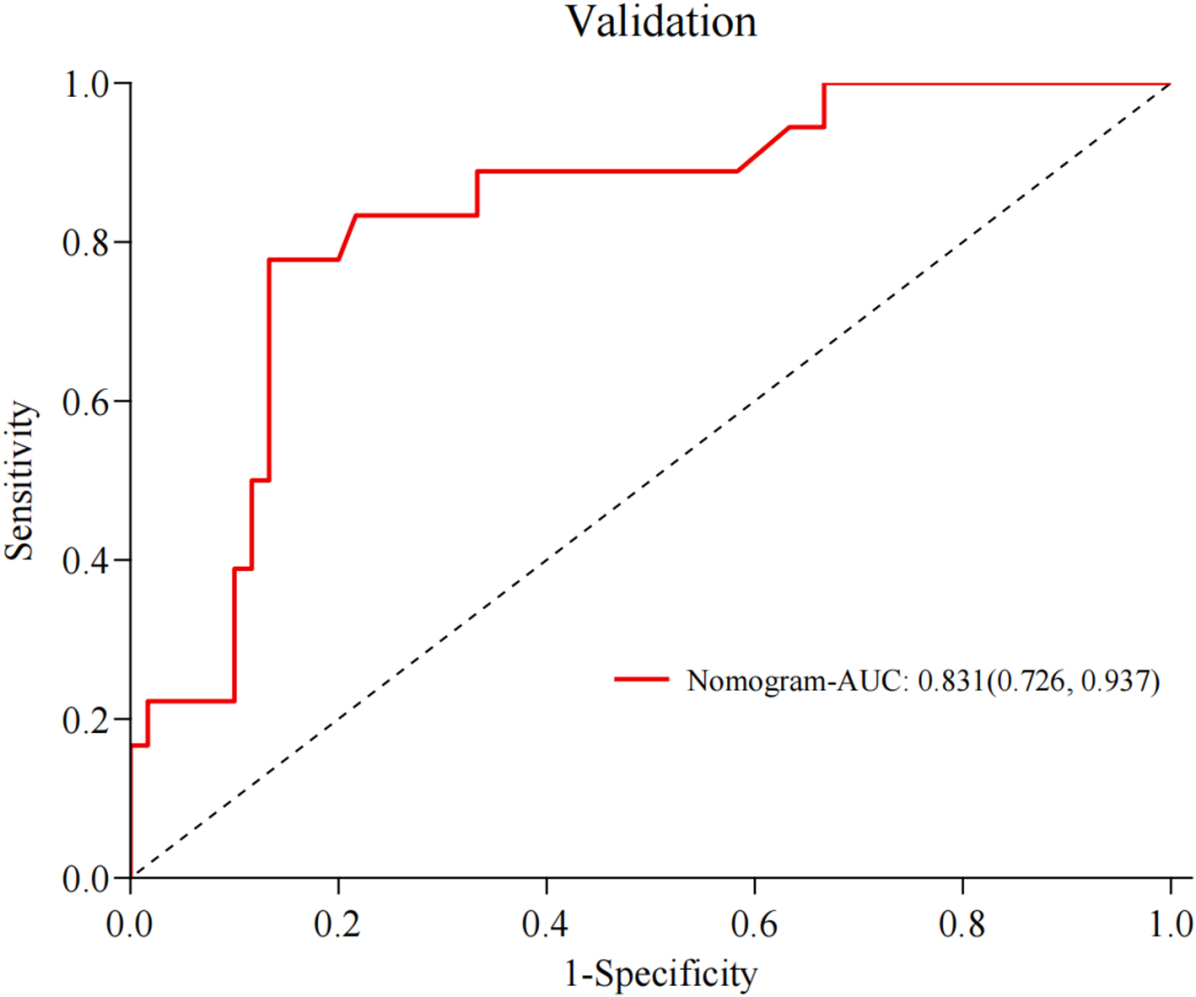
ROC curves for the validation set

We examined the discriminatory capacity of the prediction model. In the training set, the AUC was 0.793 (95% CI 0.770–0.880). The median probability of prediction accuracy was 0.810, and the maximum Youden index was 0.521. The sensitivity and specificity were 66.7% and 85.4%, respectively. Furthermore, the positive and negative predictive values were 58.3% and 89.3%, respectively. Figure 3 (ROC curves for the training set) illustrates the specific data. For the internal validation cohort, the AUC increased to 0.831 (95% CI 0.726–0.937), with a median prediction success probability of 0.846. The maximum Youden index was 0.645, with sensitivity of 77.8% and specificity of 86.7%. Lastly, the positive and negative predictive values were 63.6% and 92.9%, respectively, as illustrated in Fig. 4 (ROC curves for the validation set).

### Calibration curve for post-treatment recurrence prediction models for HSIL and differentiated vulvar lesions

**Fig. 5:**
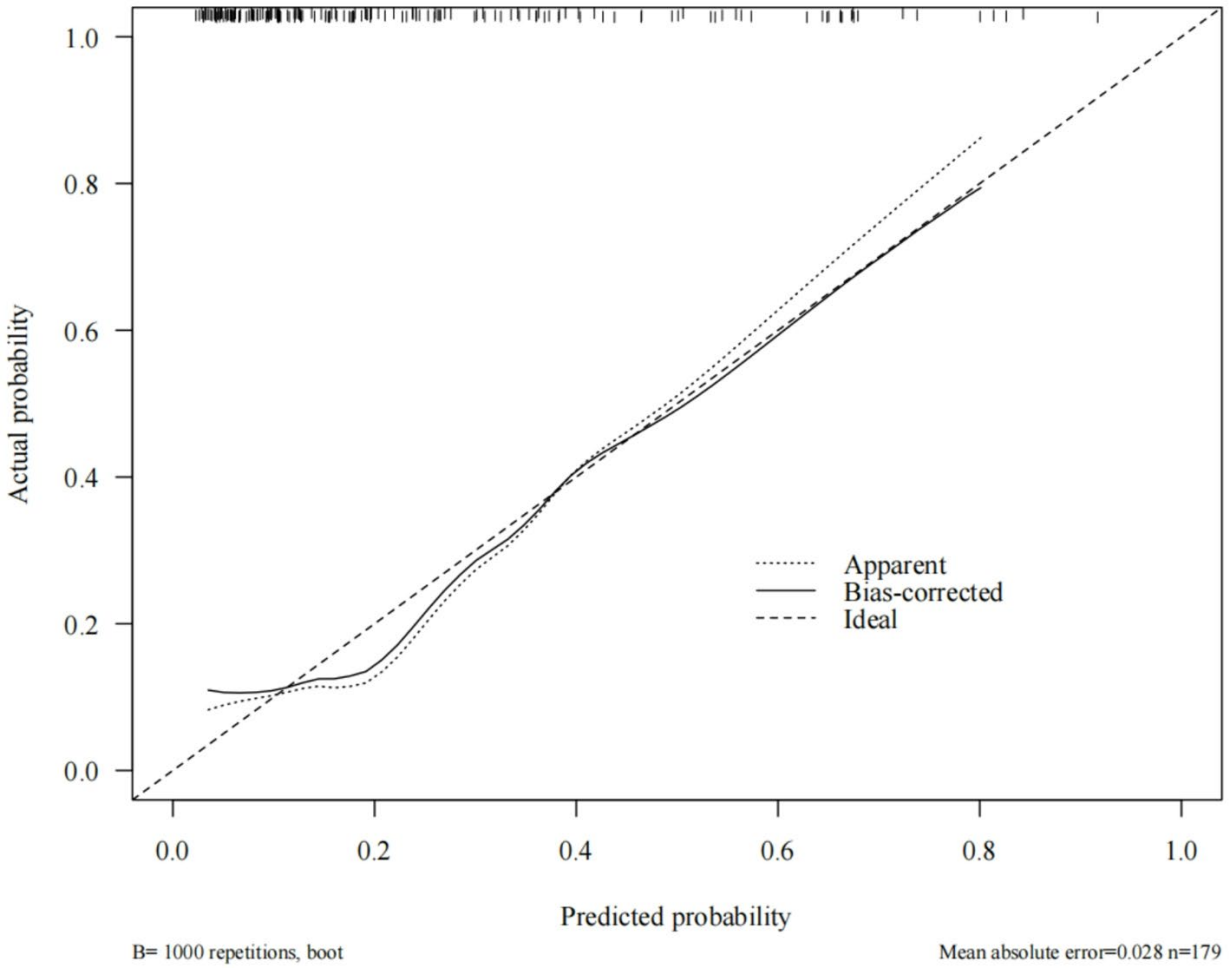
Calibration curves for the training set

**Fig. 6:**
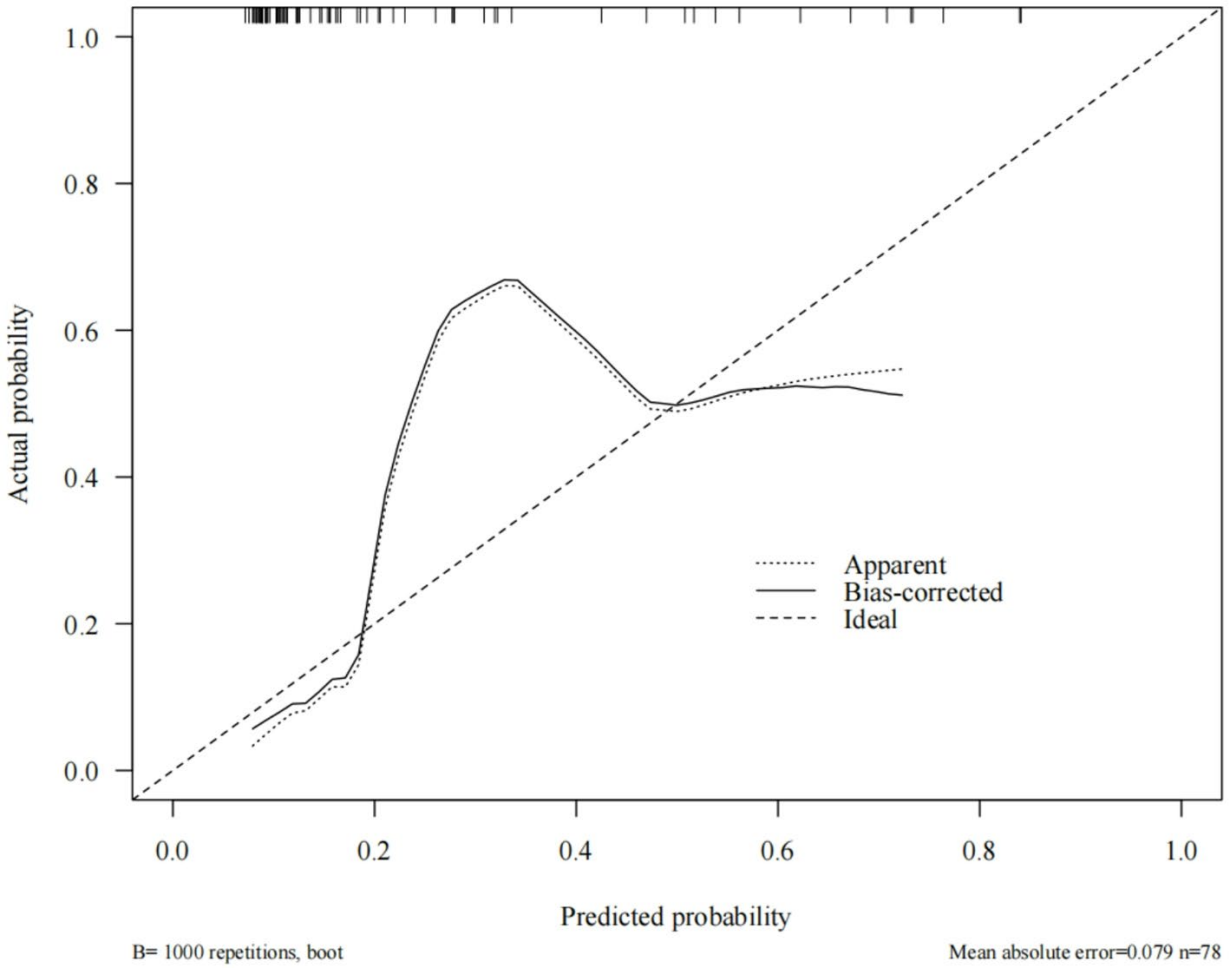
Calibration curves for the validation set

**Fig. 7:**
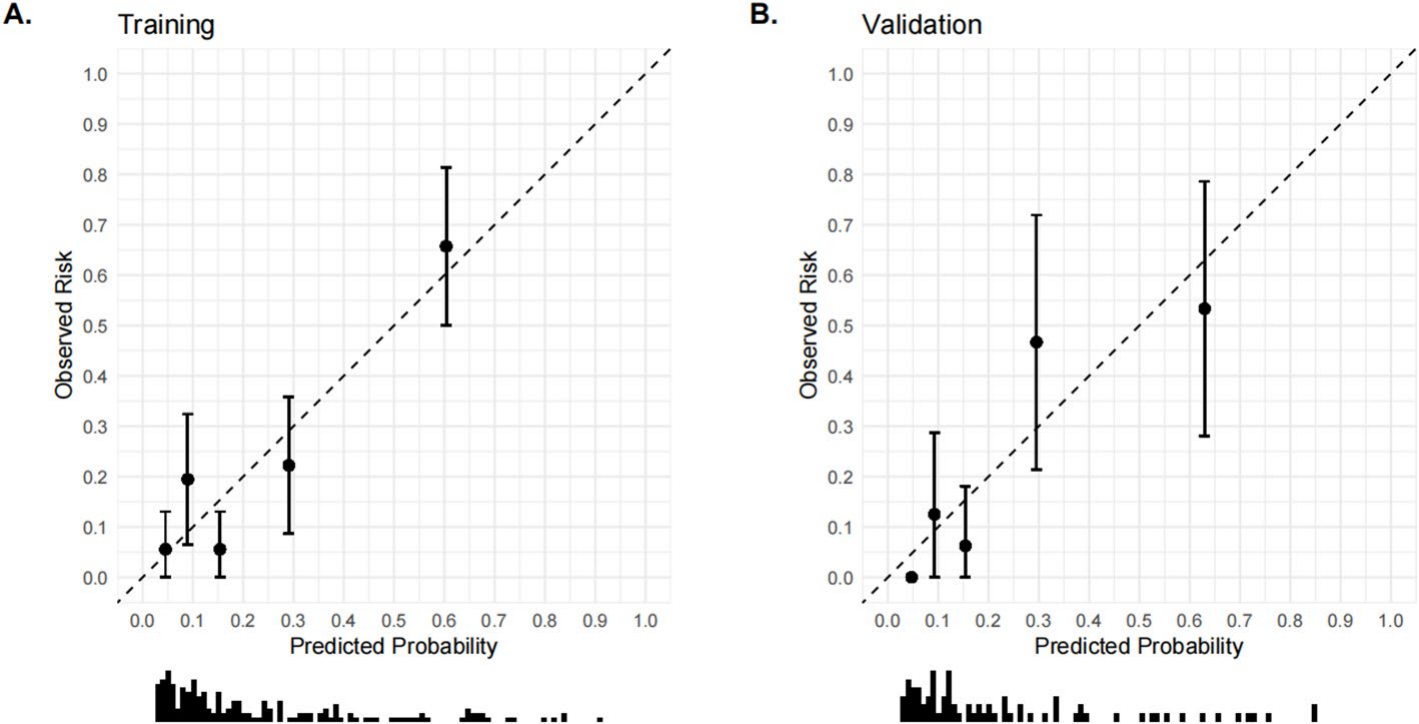
Comparison of calibration curves for training and validation sets

To generate the calibration curves, the constructed nomogram model was subjected to bootstrap sampling validation (internal bootstrap resampling, 1000 iterations). In Fig. 5, the x-axis represents the predicted probability, whereas the y-axis indicates the actual probability. The apparent curve indicates model performance without adjustments, the bias-corrected curve represents model performance after bias correction, and the ideal line indicates the perfect alignment between predicted and actual probabilities. The results were as follows. In the training set, the model’s predicted probabilities exhibited substantial concordance with the actual incidence rate, with a Kappa value of 0.496. This indicates good model accuracy. The Hosmer–Lemeshow goodness-of-fit test provided additional evidence that the model exhibits an acceptable fit within the training set (*P* = 0.069), as illustrated in Fig. 5 (Calibration curves of the training set). In the internal validation set, the model’s predicted probabilities were consistent with the actual occurrences, yielding a Kappa value of 0.598, which also indicates good accuracy. The Hosmer–Lemeshow test also exhibited an acceptable fit for the validation set model (*P* = 0.086). Detailed results are illustrated in Fig. 6 (Calibration curves of the validation set). In contrast, the AUC variation rate between the two groups was minimal, suggesting no phenomenon of overfitting. In addition, the difference between the predicted probability and observed ratio was not statistically significant, confirming the excellent calibration ability of the model. Figure 7 (Comparison of the calibration curves for the training and validation sets) illustrates the specific comparisons.

### Clinical DCA for post-treatment recurrence prediction models for HSIL and differentiated vulvar lesions

**Fig. 8:**
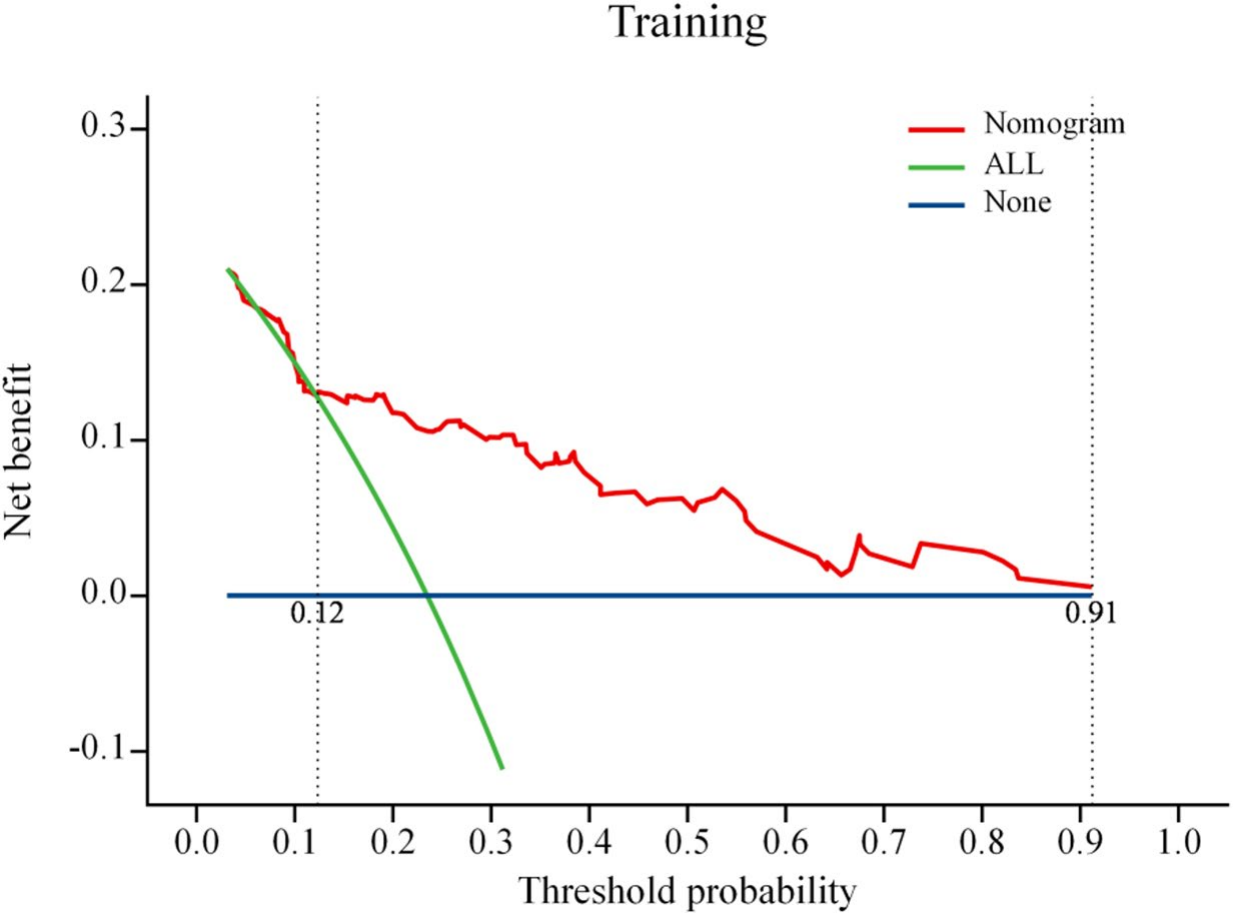
DCA curve for the training set

**Fig. 9:**
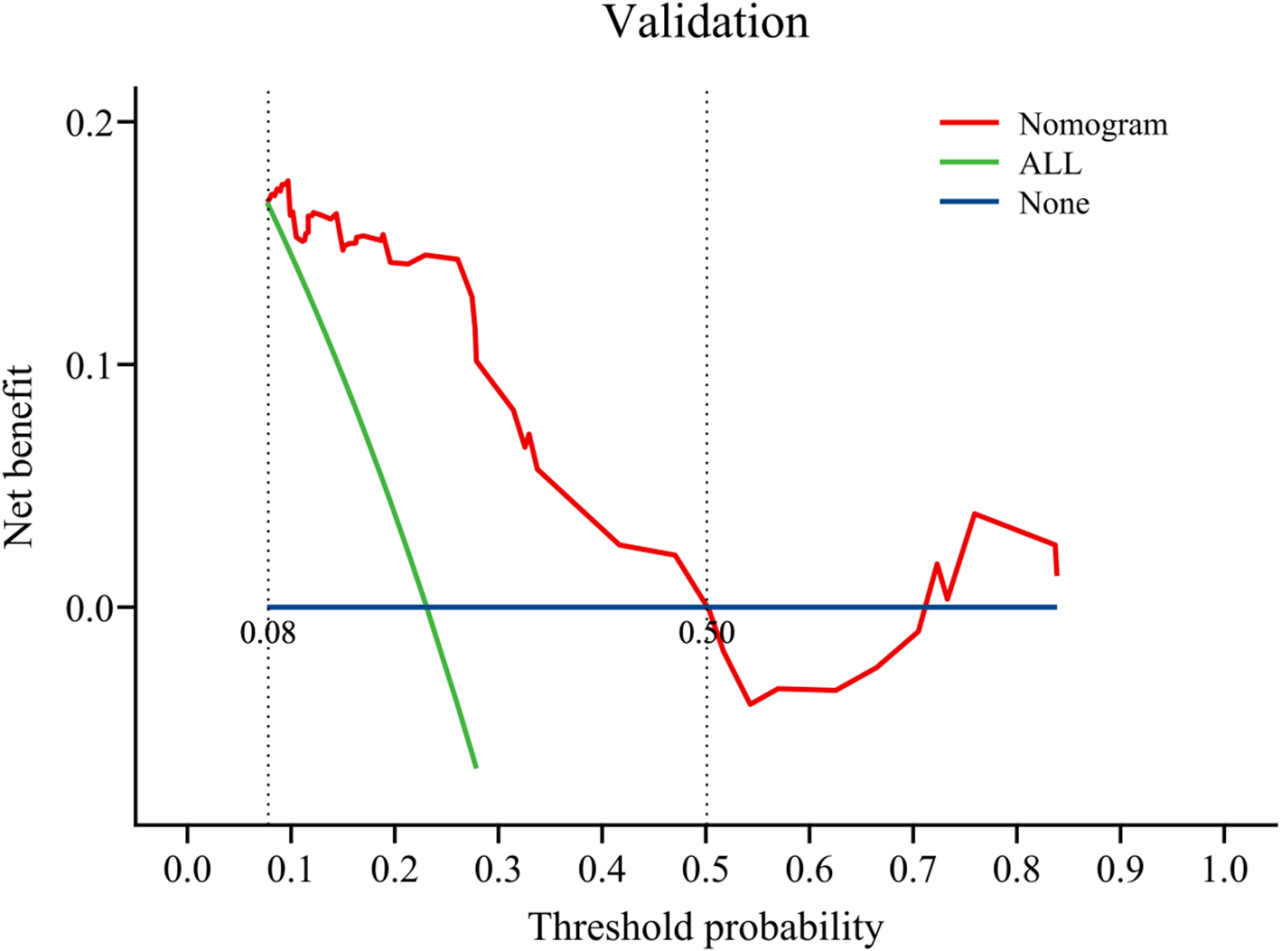
DCA curve for the validation set

By calculating the net benefit of the model, a sophisticated decision curve was constructed to rigorously evaluate the clinical utility and effectiveness of the prediction model (see Figs. 8 and 9 for the training dataset and internal validation dataset, respectively). In Fig. 8 (DCA curve for the training set) and Fig. 9 (DCA curve for the validation set), the “None” line represents the benefit curve when the patient did not receive any treatment. In contrast, the horizontal line at 0 indicates the net benefit of zero when the patients did not receive any treatment. The “ALL” line corresponds to the clinical net benefit curve derived when every patient received treatment without any discrimination. The red curve represents the overall advantage of treating the patients based on the risk thresholds defined by the developed prediction model. As illustrated in the figure, the model demonstrates a positive net benefit when the training set operates within a risk threshold probability range of 0.12 to 0.92, and the validation set falls within the corresponding range of 0.08 to 0.50—revealing its robust clinical or practical utility across diverse probabilistic scenarios. Interventions guided by the model yielded better results than both treating all patients and not treating any patients.

## Discussion

Among the malignant tumors of the female reproductive tract, vulvar cancer exhibits a relatively low incidence; however, it has an alarmingly high mortality rate and unfavorable prognosis. In the past few years, the occurrence of this cancer type has been notably increasing. Based on its etiology, vulvar cancer can be broadly categorized into two types: HPV- and non-HPV-related types. Both types differ in terms of clinical characteristics and prognosis. Owing to the lack of distinct symptoms and limited diagnostic specificity, it is often challenging to differentiate VIN from inflammation without early pathological biopsy of the lesions. Subsequently, VIN is frequently misdiagnosed by clinicians or self-diagnosed as common benign conditions such as inflammation or rash. This results in delayed follow-up and treatment. Furthermore, owing to its anatomical location and subtle presentation, VIN is easily overlooked and mistaken for inflammation. Patients frequently exhibit reluctance to seek medical care owing to embarrassment, further delaying diagnosis and treatment. While epidemiological research suggests an increasing prevalence among younger women, older women still represent the majority of the cases [18]. Chinese elderly women tend to have low willingness to seek medical care, difficulty with follow-up, and frequent refusal of follow-up appointments. Therefore, in the context of early screening and treatment of VIN, particular attention should be paid to recurrence, especially in high-risk populations that require close follow-up. In the present study, we retrospectively analyzed patient data, conducted long-term follow-up on post-treatment prognosis, monitored recurrence rates, and constructed a prediction model specifically tailored for Chinese women to improve the efficiency of follow-up care provided by Chinese gynecologists.

In this study, univariate logistic analysis revealed a positive correlation between age and menopause; however, multivariate logistic analysis did not reveal a clear association between menopause and recurrence. Considering that the decline in ovarian hormone levels post-menopause does not significantly affect this disease, elderly individuals remain the primary focus owing to their higher recurrence risk. Evidence indicates that with advancing age, prolonged disease duration, and aging-induced decline in immune surveillance, elderly women are more prone to recurrence. Therefore, clinicians should pay close attention to this group. Age is a high-risk factor, consistent with the findings of most studies [19, 20], which enhances the credibility of the results. While the correlation between smoking history and recurrence risk was marginal in univariate logistic analysis, a positive correlation was noted in multivariate logistic analysis. The inclusion of smoking history in the prediction model may explain this discrepancy. Owing to traditional Chinese cultural customs, fewer women smoke in China, particularly elderly women. Although smoking is a recognized contributing factor for VIN, studies have not yet explored its correlation with recurrence. In the present study, we aimed to raise clinicians’ awareness regarding smoking patients. We noted an association between immunodeficiency and the risk of recurrence; this finding is consistent with those of a previous study [21]. Interestingly, there was no correlation between autoimmune diseases and recurrence; however, a positive correlation was found between immunosuppressants and recurrence. Immune escape may only occur when immune function is compromised, leading to an increased risk of recurrence. In contrast, strong immune responses do not impair the ability to clear abnormal cells, thereby not increasing the risk of recurrence. Similarly, while many studies suggest that high BMI and type 2 diabetes increase chronic inflammation and immune dysfunction, thereby increasing the risk of various malignant tumors and precancerous lesions [22–24], we did not identify such a correlation. HPV typing studies have confirmed that HPV type 16 is strongly correlated with recurrence, whereas other high-risk HPV infections exhibit no significant correlation. In univariate logistic analysis, low-risk HPV infections were found to be negatively correlated; however, this correlation lacked significance in multivariate logistic analysis. Considering that the predominant high-risk HPV types in Chinese women are HPV16 and HPV18, early screening focused on these two types, introducing potential case selection bias. Persistent high-risk HPV infection continues to be a predominant risk factor for VIN onset [25, 26]; its increasing incidence correlates with HPV infection [27]. However, in this study, we observed that only HPV16 was associated with recurrence. This underscores the need to prioritize HPV16-infected patients as a key group for follow-up. Histopathological studies have revealed that dVIN carries a higher recurrence risk, consistent with the findings of most studies [28, 29]. Furthermore, it exhibits a higher probability of progressing to malignancy compared with HPV-related pathways [30]. Effective management and careful monitoring of conditions associated with dVIN, including VLS and lichen planus, can considerably decrease the likelihood of progression [6, 11]. However, the immunohistochemical markers p53 and Ki67 were not associated with recurrence, contradicting the findings of pathologists [29]. Due to the insufficient sample size, further verification through large multicenter studies is warranted. We did not observe differences in recurrence risk across all currently recommended treatments; this suggests that treatment selection can be based on local treatment capabilities and resources. In surgical treatment, the status of the incisal margin is an important concern for gynecologists. While a previous study in Southern California revealed an association [31], univariate logistic analysis in this study reached the same conclusion; however, multivariate logistic analysis excluded this factor as a significant predictor. Therefore, if clinical examination confirms no residual lesion after surgical resection with a positive incisal margin, follow-up and not resection is recommended. This study represents the pioneering effort to determine the application of interferon as an adjuvant therapy for preventing recurrence after the management of vulvar HSIL. Unfortunately, interferon therapy did not exhibit a preventive effect, highlighting the need for larger sample evaluations of interferon-based immunotherapy.In addition, lesion size, number of lesions, presence of cervical disease, and visible vulvar lesions or symptoms were included in the analysis; however, none of these factors exhibited a correlation with recurrence. Based on our resutls, clinicians should focus on elderly women, those with a smoking history, immunocompromised individuals, patients with HPV16 infections, and patients with dVIN to enhance follow-up efficiency. However, given the limited sample size and lack of effect, more data needs to be collected.

In the present study, we identified age, smoking history, immunosuppression, HPV16 infection, and histopathological characteristics as key differential factors for the post-treatment recurrence of HSIL and differentiated vulvar lesions. Therefore, these factors were included in the regression analysis to construct a prediction model. The model’s predictive capabilities were thoroughly assessed, with the training and validation sets yielding AUC values of 0.793 and 0.831, respectively.The model’s discriminatory ability falls short of “excellent” performance (AUC > 0.90), suggesting room for improvement in risk prediction accuracy in the future. Meanwhile, a positive predictive value (PPV) of 58–64% implies a non-negligible false-positive rate in low-prevalence populations, potentially leading to overdiagnosis or unnecessary interventions. The model’s clinical utility is confined to a limited risk threshold range, restricting generalizability to broader populations. These findings suggest the exceptional precision of the model in predicting HSIL recurrence and distinguishing vulvar lesions after treatment. Calibration curve analysis revealed that the working and bias-corrected curves followed the same trend and were similar to the ideal curve, suggesting good calibration of the nomogram. In addition, the Hosmer–Lemeshow test results (*P* = 0.069 for the training set and *P* = 0.086 for the internal validation set) provided additional evidence of the high goodness-of-fit of the prediction model, with no statistically significant difference compared with the ideal model. Moreover, clinical DCA revealed that, under identical threshold probabilities, the predictions made using this model offered a significantly higher net benefit compared with extreme approaches, thereby reinforcing its strong clinical relevance. Collectively, these validations confirm the development of an effective prediction model for post-treatment recurrence of HSIL and differentiated vulvar lesions. In the future, this prediction model could be integrated into software applications, allowing clinicians to conveniently evaluate individual patients via web-based platforms or mobile applications.

## Study limitations

 (1) Because of the low prevalence of DEVIL and VAAD, patients with these conditions were not included in this study. Subsequently, the recurrence risk of these two non-HPV-associated VINs could not be assessed. ‌Exclusion of Positive Surgical Margins (PSM)‌: The final model omitted PSM, a well-established prognostic factor for recurrence, despite its significance in univariate analysis. This exclusion may have artificially reduced the apparent risk associated with surgical quality, potentially misleading clinical decision-making. Future studies should prioritize its inclusion to enhance model validity.‌Binary Outcome Measurement‌: The use of a binary recurrence outcome (yes/no within ≥ 2 years) fails to capture temporal dynamics of risk. A time-to-event analysis (e.g., Cox proportional hazards model with Kaplan-Meier curves) would better reflect the evolving nature of recurrence risk and improve prognostic accuracy. ‌Lack of Subtype Stratification‌: The model did not differentiate between vaginal high-grade squamous intraepithelial lesion (VHSIL) and differentiated vulvar intraepithelial neoplasia (dVIN), despite their distinct biological behaviors and risk profiles. This omission may obscure heterogeneous recurrence patterns. Stratified analyses or explicit acknowledgment of this limitation are warranted in future research. (2) The relatively small sample size may lead to some bias. Furthermore, this is a single-center study that was conducted in a local area. Therefore, it excludes statistical analysis of differences in geography, ethnicity, race, or dietary habits. In addition, based on regional nutritional habits and economic income, the BMI of the female participants is primarily within the normal range, higher than that in impoverished areas but lower than that in high-income areas. Therefore, it is not possible to compare different body types. In addition, There are only 60 events (such as adverse health outcomes or disease cases) in the study population, and statistical models may not be able to detect meaningful associations with rare exposures such as smoking and immunosuppressive therapy. Due to demographic and cultural factors (such as higher historical smoking rates among older generations and increased use of immunosuppressive drugs for chronic diseases), these exposures are common among elderly women in China, but still uncommon at the population level. The limited sample size reduces the statistical ability to identify significant effects, especially for low prevalence exposures. This sparse data increases the risk of type II errors (false negatives), where true associations are missed due to insufficient evidence. (3) All participants are Han Chinese and there are no representatives of other ethnic groups. The homogeneity of participant selection stems from practical limitations such as recruitment channels and geographical concentration of Han population in the study area, but it introduces significant methodological limitations. Considering that all participants were recruited from the same genetic background, the universality of this model is limited. Racial groups typically exhibit different genetic, cultural, and environmental characteristics, which can affect behavioral patterns, health outcomes, and responses to intervention measures. For example, cultural customs or dietary habits unique to other races may alter the applicability of research results centered on Han Chinese samples. Therefore, the research findings may not be applicable to other populations, especially those with different demographic or socio-cultural backgrounds. This limitation emphasizes the need for future research to incorporate diverse racial representations to enhance universality and avoid potential biases in cross-cultural studies. Without this diversity, the model may overgeneralize conclusions, which could mislead policies or clinical applications in multicultural environments. Recognizing this constraint is crucial for advancing inclusive research frameworks that acknowledge the multifaceted nature of human diversity.In the future, studies with larger sample sizes are warranted to improve the reliability and generalizability of the model. Another potentially effective strategy could be to use generative methods for data augmentation. By using techniques such as rotation, scaling, and flipping, we can artificially create additional training samples to expand the dataset. (4) This was a single-center study and not an HIV treatment center. No patients who underwent organ transplants were identified. Therefore, there is a potential bias in evaluating immunosuppressive factors, necessitating future collaboration among multiple regions, centers, and disciplines to enhance the assessment of this factor. (4) Our study findings are retrospective; therefore, prospective studies are warranted to verify the clinical effectiveness of our findings. (5) Our prediction model has not undergone external validation. The model has only undergone internal validation, which inherently limits its generalizability and raises concerns about potential bias or overfitting of specific datasets and conditions studied internally. Although internal validation is valuable for initial performance evaluation, it often involves individual institutions or homogeneous patient populations, which may not accurately reflect the real-world diversity of patient demographics, clinical practices, or environmental factors. Without external validation, the robustness and reliability of the model remain uncertain, as internal evaluations often lack rigorous scrutiny from independent experts and fail to consider differences between different medical environments. Any new prediction model should undergo a comprehensive evaluation of its effectiveness before clinical application. We have collected additional clinical data to perform external validation, ensuring the universality and reproducibility of the model. This is essential for translating our research findings into clinical practice. In the future, we will further optimize the model by conducting prospective external validation and incorporating more factors to enhance its applicability and accuracy, thereby addressing the bias issue.

In summary, by combining clinical parameters, our model excels in predicting the post-treatment recurrence rate of HSIL and differentiated vulvar lesions. This model can help gynecologists identify individuals with high recurrence risk after treatment for HSIL and differentiated vulvar lesions. Furthermore, it provides a foundation for clinical decision-making and the development of targeted intervention measures. In the future, multicenter prospective validation and mechanistic studies should be conducted to support broader clinical application of the model.

## Data Availability

All data generated or analyzed during this study are included in this published article.The data is available at Ye, X. (2025). Development of a Risk Assessment Model for the Recurrence of HSIL and differentiated vulvar intraepithelial neoplasia [Data set]. Zenodo. https://doi.org/10.5281/zenodo.16417081.

## Abbreviations

HSIL: High-grade squamous intraepithelial lesions
dVIN: differentiated vulvar intraepithelial neoplasia
LASSO: Least Absolute Shrinkage and Selection Operator
HPV: Human papillomavirus
AUC: Area under the receiver operating characteristic curve
CI: Confidence intervals
VSCC: Vulvar squamous cell carcinoma
VIN: Vulvar intraepithelial neoplasia
VLS: Vulvar lichen sclerosus
NNEDV: Non-neoplastic epithelial disorders of the vulva
DEVIL: Differentiated exophytic vulvar intraepithelial lesion
VAAD: Vulvar acanthosis with altered differentiation
WHO: World Health Organization
PDT: Photodynamic therapy
CO_2_: Carbon dioxide
BMI: Body mass index
p53: protein 53
Ki67: Nuclear proliferation-associated antigen
SPSS: Statistical Product and Service Solutions
R software: R Programming Language software
P25: Twenty-fifth percentile
P75: Seventy-fifth percentile
DCA: Decision curve analysis
HL: Hosmer-Lemeshow
β: Regression coefficient
S.E.: Standard error
OR: Odds ratios
PPV: Positive predictive value
NPV: Negative predictive value
